# Mechanisms of Edible Bird's Nest Extract-Induced Proliferation of Human Adipose-Derived Stem Cells

**DOI:** 10.1155/2012/797520

**Published:** 2011-11-01

**Authors:** Kyung-Baeg Roh, Jienny Lee, Young-Soo Kim, Junho Park, Jang-Hyun Kim, Jongsung Lee, Deokhoon Park

**Affiliations:** ^1^Biospectrum Life Science Institute, 442-13 Sangdaewon-Dong, Seongnam City, Gyeonggi Do 462-807, Republic of Korea; ^2^Skincure Life Science Institute, 66 Jejudaehakno, Jeju City, Jeju Special Self-Governing Province 690-756, Republic of Korea; ^3^Dermiskin Life Science Institute, 44-9 Cheongho Ri, Pyeongtaek City, Gyunggi Do 451-862, Republic of Korea

## Abstract

Although edible bird's nest (EBN) has been shown to potentiate mitogenic responses, scientific evidence of its efficacy is still limited. In addition, human adipose-derived stem cells (hADSCs) are increasingly accepted as a source for stem cell therapy. Therefore, the aim of this study was to investigate the effects of the EBN extract (EBNE) on the proliferation of hADSCs and its action mechanisms. We found that EBNE strongly promoted the proliferation of hADSCs. In addition, EBNE-induced proliferation was found to be mediated through the production of IL-6 and VEGF, which was induced by activation of AP-1 and NF-**κ**B. Specially, we found that production of IL-6 and VEGF was induced by EBNE. In addition, EBNE-induced production of IL-6 and VEGF was inhibited by PD98059 (a p44/42 MAPK inhibitor), SB203580 (a p38 MAPK inhibitor), and PDTC (a NF-**κ**B inhibitor), but not SP600125 (a JNK inhibitor). Similarly, EBNE-induced proliferation of hADSCs was also attenuated by PD98059, SB203580, and PDTC but not SP600125. Taken together, these findings suggest that the EBNE-induced proliferation of hADSCs primarily occurs through increased expression of IL-6 and VEGF genes, which is mediated by the activation of NF-**κ**B and AP-1 through p44/42 MAPK and p38 MAPK.

## 1. Introduction

Edible bird's nest (EBN) is the nest of the swift and is constructed with salivary glue, which is a cementing substance, and may incorporate other materials such as vegetation or feathers. Although EBN mainly contains carbohydrates, amino acids, and mineral salts, its major ingredients are glycoproteins [1]. Due to its nutritious and medical properties, EBN has been deemed a precious food tonic in Chinese community ever since the Tang (907 AD) and Sung (960–1279 AD) dynasties [2]. Despite the long history of using EBN for medicinal purpose, there have only been a limited number of scientific reports on the health benefits of EBN. Recently, EBN has been found to potentiate mitogenic response of human peripheral blood monocytes [3] and stimulate DNA synthesis in 3T3 fibroblasts [4].

Growth factors such as epidermal growth factor (EGF) and vascular endothelial growth factor (VEGF) and cytokines such as interleukin-6 (IL-6) play an important role in cellular communication. As intercellular mediators, these molecules regulate survival, growth, differentiation, and effector functions of cells [5–8]. In the case of stem cells, the IL-6 family of cytokines has been known to be involved in the maintenance of both embryonic and adult stem cells [9, 10]. In addition, IL-6 was reported to increase the proliferation of placenta-derived multipotent mesenchymal stem cells (MSCs) [11]. Rehman et al. also demonstrated that human adipose stromal cells secreted angiogenic and antiapoptotic growth factors, such as VEGF, and hepatocyte growth factor (HGF), in response to hypoxia and that conditioned media containing these growth factors increased cell growth and suppressed cell apoptosis [12]. 

MSCs are adult progenitor cells isolated primarily from bone marrow or adipose tissue and can be rapidly expanded in large numbers. MSCs are capable of differentiating into several cell lineages, including osteoblasts, chondrocytes, adipocytes, and myoblasts [13, 14]. In addition, MSCs were reported to be involved in the repair of injured tissue through the secretion of cytokines, chemokines, and growth factors [15–17]. Due to these properties, MSCs are becoming a promising source of cells for various clinical applications [18].

In the present study, we demonstrated that EBNE promoted the proliferation of hADSCs and that its effects were mediated via the expression of IL-6 and VEGF through the activation of NF-*κ*B and AP-1, which were activated by p44/42 MAPK and p38 MAPK.

## 2. Material and Methods

### 2.1. Preparation of Edible Bird's Nest Extract (EBNE)

EBN was purchased from xiamen xiang long yan trade Co., Ltd (Xiamen, China). The EBN was dried for 24 h at 50°C and then ground. The ground EBN was kept in distilled water at 50°C for 30 min. The suspension was treated with enzyme, Protamex (final concentration of 1% in water, Novozymes, Bagsvaerd, Denmark) at 50°C for 24 h and then heated at 100°C for 15 min to inactivate the enzyme. The supernatant was obtained after centrifugation for 20 min at 3,000 ×g and subjected to ultrafiltration using a 3,000 Da cut-off membrane. The filtrated sample containing compounds with a molecular weight lower than 3,000 Da was lyophilized and then dissolved in Dulbecco's modified Eagle's medium (DMEM, WelGENE, Daegu, Korea). Amino acid distribution (mg/g) of EBNE was measured (Table 1).

### 2.2. Chemicals and Antibodies

MAPK inhibitors (PD98059, SB203580, SP600125, PDTC), antibodies (p44/42, phospho-p44/42 (Thr202/Tyr204), p38, phospho-p38 (Thr180/Tyr182) MAPK, stress-activated protein kinase/C-Jun N-terminal kinse (SAPK/JNK), phospho-SAPK/JNK (Thr183/Tyr185)), and phospho-I*κ*B-*α* were all purchased from Cell Signaling Technology, Inc (Beverly, Mass, USA).

### 2.3. Cell Culture

hADSCs were originally purchased from Invitrogen (Carlsbad, Calif, USA) and cultured at 37°C under 5% CO_2_ in MesenPRO RS medium (GIBCO, Carlsbad, Calif, USA) with Growth supplement (GIBCO, Carlsbad, Calif, USA). Normal human fibroblasts (NHFs) were obtained from Amorepacific, Inc. (Yongin, Korea) and cultured in DMEM containing high-glucose levels, 10% fetal bovine serum (FBS, GIBCO, Carlsbad, Calif, USA) and 1% penicillin/streptomycin (Invitrogen, Carlsbad, Calif, USA) at 37°C under 5% CO_2_. Hep-3B (KCLB No. 88064) and MCF-7 (KCLB No. 30022) were purchased from the Korean Cell Line Bank (Seoul, Korea) and cultured in DMEM containing high-glucose levels, 10% FBS and 1% penicillin/streptomycin at 37°C under 5% CO_2_.

### 2.4. Cell Proliferation

Cell proliferation was measured using the MTT (3-[4,5-Dimethyl-2-thiazolyl]-2,5-diphenyl-2H-tetrazolium bromide, USB Corp, Cleveland, Ohio, USA) and assay. Cells (1 × 10^5^) were plated in triplicate wells of 6 well-plates and incubated overnight. The cells were then treated with 2,000 ppm EBNE for 60 h under serum-free condition. After treatment, MTT reagent (1 mg/mL) was added to each well. Cells were then incubated for 3 h with MTT. Medium was then removed and cells were solubilized with dimethylsulphoxide (DMSO). Absorbance was measured at a wavelength of 570 nm using a spectrophotometer. As a positive controls, cells were cultured in control medium (DMEM + 15% FBS: hADSCs, Hep-3B, MCF-7 and NHFs; DMEM + 10% FBS).

### 2.5. Cell Proliferation

Cell proliferation was measured using the Click-iT EdU Cytometry Assay Kit (Invitrogen, Carlsbad, Calif, USA) according to the manufacturer's instructions. 1 × 10^5^ cells were seeded in 60 mm dishes and incubated overnight. The cells were then treated with 2,000 ppm EBNE for 60 h under serum-free conditions. The cells were then labeled with EdU (10 *μ*M) for 3 h, fixed for 15 min with 4% paraformaldehyde and permeabilized for 5 min at room temperature using Triton X-100 solution. The fixed cells were stained with the Click-iT reaction mixture for 30 min at room temperature, and then analyzed using a C6 Flow Cytometer (Accuri Cytometers, Ann Arbor, Mich, USA).

### 2.6. Western Blotting

Western blotting was performed to measure the levels of proteins associated with the MAPK pathways. The protein extracts (40 *μ*g/lane) were loaded on a NuPAGE Novex 10% Bis-Tris Gel (Invitrogen, Carlsbad, Calif, USA) and transferred to a nitrocellulose membrane. The membranes were blocked with 5% bovine serum albumin (BSA) for 1 h and then incubated with primary antibodies, followed by incubation with horseradish peroxidase-conjugated anti-mouse or rabbit IgG secondary antibody and detected using PowerOpti-ECL Western blotting Detection reagent (Anigen, Hwaseong, Korea).

### 2.7. ELISA

IL-6 and VEGF concentrations were quantified in culture supernatants from hADSCs proliferation assays using a commercially available ELISA kit (R&D systems, Minneapolis, Minn, USA). Cell culture supernatants were collected 60 h after treatment with 2,000 ppm EBNE and assayed for IL-6 and VEGF. The standard curve was linearized and subjected to regression analysis. The IL-6 and VEGF concentration of the unknown samples was calculated using the standard curve. All samples and standards were measured in duplicate.

### 2.8. Cytokine Profile

For quantitative analysis of cytokines, supernatants of hADSCs cultured with 2,000 ppm EBNE in serum-free DMEM for 60 h were analyzed using a fluorescent bead immunoassay, the FlowCytomix human basic kit (eBioscience, San Diego, Calif, USA), following the manufacturer's instructions. Briefly, standards and cell supernatants were placed in 96-well filter plates, and then coated beads and biotinylated detection beads were added to all wells and incubated for 2 h. The mixture was then incubated with PE-labelled streptavidin for 1 h and measured using flow cytometry. Data were analyzed using the FlowCytomixPro software (eBioscience, San Diego, Calif, USA).

### 2.9. RNA Extraction and Real-Time Quantitative Reverse Transcription-PCR (RT-qPCR)

Total RNA extraction was carried out using the RNeasy kit (QIAGEN, Hilden, Germany) for isolation of total RNA according to the manufacturer's instructions. The first cDNA was synthesized using a PrimeScript 1st strand cDNA synthesis kit (TAKARA, Tokyo, Japan) according to the manufacturer's instructions. To quantify cytokine gene expression, fluorescence real-time PCR was performed using the double-stranded DNA dye, SYBR Green (Applied Biosystems, Foster city, Calif, USA). Primer pairs for hIL-6 (sense, 5′-GGT ACA TCC TCG ACG GCA TCT-3′; antisense, 5′-GTG CCT CTT TGC TGC TTT CAC-3′), hVEGF (sense, 5′-CTA CCT CCA TGC CAA GT-3′; antisense, 5′-GCA GTA GCT GCG CTG ATA GA-3′), and the control hGAPDH (sense, 5′-TGC ACC ACC AAC TGC TTA GC-3′; antisense, 5′-GGC ATG GAC TGT GGT CAT GAG-3′) were used to detect the target gene transcripts. SYBR Green analysis was performed on an ABI PRISM 7300 system (Applied Biosystems, Foster city, Calif, USA) according to the manufacturer's instructions. All samples were analyzed in triplicate, and the levels of the detected mRNAs were normalized to control hGAPDH values. The normalized data were used to quantify the relative levels of a given mRNA according to the ΔCt analysis.

### 2.10. Transient Transfection and Luciferase Assay

hADSCs were transfected with AP-1, CRE, and NF-*κ*B-Luc reporter, along with Renilla luciferase expression vector driven by thymidine kinase promoter (Promega, *Madison*, Wis, USA) using SuperFect transfection reagent (Qiagen, Valencia, Calif, USA). After incubation for 24 h, cells were incubated in the presence or absence of EBNE for 24 h. The cells were then harvested and lysed. Supernatants were assayed for luciferase activity. Luciferase activity was determined using a Dual Luciferase Assay system (Promega, *Madison*, Wis, USA) and a LB953 luminometer (Berthold, Germany). The activity was expressed as a ratio of the NF-*κ*B-dependent firefly luciferase activity to the control thymidine kinase Renilla luciferase activity.

### 2.11. Immunophenotyping

10 surface markers were analyzed by flow cytometry. Markers of hMSCs (CD11b-PE, CD14-APC, CD19-PE, CD34-PE, CD44-PE, CD45-APC, CD73-APC, CD90-APC, CD105-PE, and HLA-DR-APC) and negative control isotypes (PE-conjugated IgG1 k, and APC-conjugated IgG1 k, PE-conjugated IgG2b k) were all purchased from eBioscience, Inc. Cells (5 × 10^5^) were seeded in 100 mm dishes and incubated overnight. The cells were then treated with 2,000 ppm EBNE for 60 h under serum-free conditions. After 60 h, the cells were stained with the indicated antibodies for 20 min at room temperature and then analyzed using flow cytometry.

### 2.12. Statistical Analysis

All data were expressed as the means ± SD. Differences between the control and the treated group were evaluated by a Student's *t-*test using the Statview software (Abacus Concepts, Cary, NC, USA). For all analyses, a *P* < 0.05 was considered statistically significant.

## 3. Results

### 3.1. Effects of EBNE on Cell Proliferation of hADSCs

**Figure 1:**
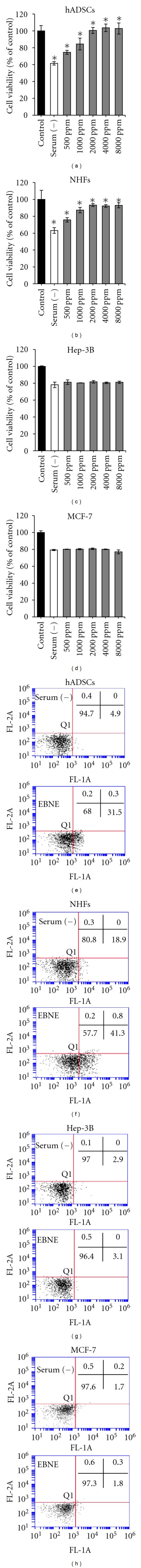
Effects of EBNE on the proliferation of hADSCs, NHFs, Hep-3B, and MCF-7. (a)–(d) Cells were cultured in serum-free DMEM in the presence or absence of the indicated concentrations of EBNE. Cell proliferation was then determined using the MTT assay. (a), (b), (c), and (d) represent the proliferation of hADSCs, NHFs, Hep-3B, and MCF-7, respectively. For the positive control, cells were cultured in control medium (DMEM + 15% FBS: hADSCs, Hep-3B, MCF-7 and NHFs; DMEM + 10% FBS). Values are represented as percentage compared with the control. Results are shown as mean ± SD (*n* = 3). *P < 0.01 versus serum-free control. Data are representative of at least three independent experiments. (e)–(h) Cell proliferation was determined using Click-iT EdU flow cytometry. (e), (f), (g), and (h) represent the proliferation of hADSCs, NHFs, Hep-3B, and MCF-7, respectively. Serum (−): serum-free, EBNE: 2,000 ppm of EBNE. Data are representative of at least three independent experiments.

We first examined the effects of EBNE on cell proliferation of hADSCs and NHFs under serum-free conditions. After the cells were treated with various concentrations of EBNE (500 ppm, 1,000 ppm, 2,000 ppm, 4,000 ppm, 8,000 ppm), cell proliferation was measured using the MTT assay. As shown in Figures 1(a) and 1(b), EBNE concentration dependently increased cell proliferation, which reached high levels at 2,000 ppm. Specifically, while EBNE induced a 34% and 38% increase in the cell proliferation rate of hADSCs and NHFs, respectively, compared to the untreated serum-free group, no observable effects on the two human cancer cell lines tested (MCF-7, human breast cancer and Hep2B, human liver cancer) were observed (Figures 1(c) and 1(d)). These suggest that the EBNE effects are limited to normal cells and do not affect transformed cell lines. The cell-proliferating effects of EBNE were further confirmed using the Click-iT EdU Flow cytometry assay. Similar to the results presented in Figures 1(a)~1(d), while the EBNE-treated cells were shifted to the right when compared to the untreated hADSCs and NHFs (Figures 1(e) and 1(f)), MCF-7 and Hep2B cells were not affected by EBNE (Figures 1(g) and 1(h)). These results indicate that EBNE contributed to the increased proliferation of hADSCs and NHFs, but not human cancer cell lines.

### 3.2. Effects of EBNE on Expression Profile of Cytokine Genes

**Figure 2:**
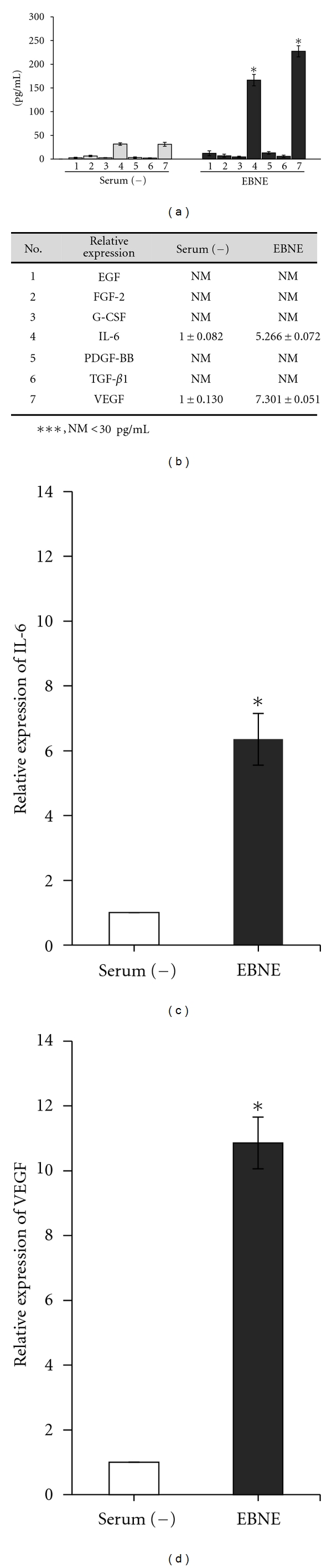
Cytokine profile analysis of hADSCs treated with EBNE. hADSCs were cultured in the presence of 2,000 ppm EBNE for 60 h. EGF (column 1), FGF-2 (column 2), G-CSF (column 3), IL-6 (column 4), PDGF-BB (column 5), TGF-*β*1 (column 6), and VEGF (column 7). (a, b) hADSCs were cultured in serum-free DMEM in the presence or absence of 2,000 ppm EBNE, and the concentration of produced cytokines (pg/mL) was measured using a flow cytometric bead assay. Results are shown as mean ± SD in (a) (*n* = 2). *P < 0.05 versus serum-free controls. ***NM indicates that treatment with EBNE-induced production of <30 pg/mL of cytokines. Data are representative of at least three independent experiments. (c, d) mRNA levels of IL-6 (c) and VEGF (d) were measured using real-time PCR. Results are shown as mean ± SD (*n* = 3). *P < 0.05 versus serum-free controls. Serum (−): serum free, EBNE: 2,000 ppm of EBNE.

MTT and flow cytometry assays indicated that EBNE was involved in the proliferation of hADSCs and HNFs. Therefore, the effects of EBNE on cytokine expression in hADSCs were examined using a human fluorescent bead immunoassay in order to elucidate the mechanisms behind EBNE-induced proliferation. Epidermal growth factor (EGF), fibroblast growth factor-2 (FGF-2), granulocyte colony-stimulating factor (G-CSF), interleukin-6 (IL-6), platelet-derived growth factor BB (PDGF-BB), transforming growth factor-beta1 (TGF-*β*1), and vascular endothelial growth factor (VEGF) were included in this study. The levels of expression of IL-6 (166.5 pg/mL ± 12.02) and VEGF (227.5 pg/mL ± 11.66) were significantly high among these cytokines (Figures 2(a) and 2(b)). However, no significant changes in the other cytokines were observed in response to treatment with EBNE in hADSCs (Figures 2(a) and 2(b)). In addition, EBNE-induced expression of IL-6 and VEGF was also further confirmed using a real-time PCR assay. As shown in Figure 2(c), the mRNA levels of IL-6 and VEGF were significantly increased upon treatment with EBNE (Figures 2(c) and 2(d)). These results suggest that IL-6 and VEGF play a critical role in the EBNE-induced proliferation of hADSCs.

### 3.3. AP-1 and NF-*κ*B Signaling Are Involved in the EBNE-Induced Expression of IL-6 and VEGF Genes

**Figure 3:**
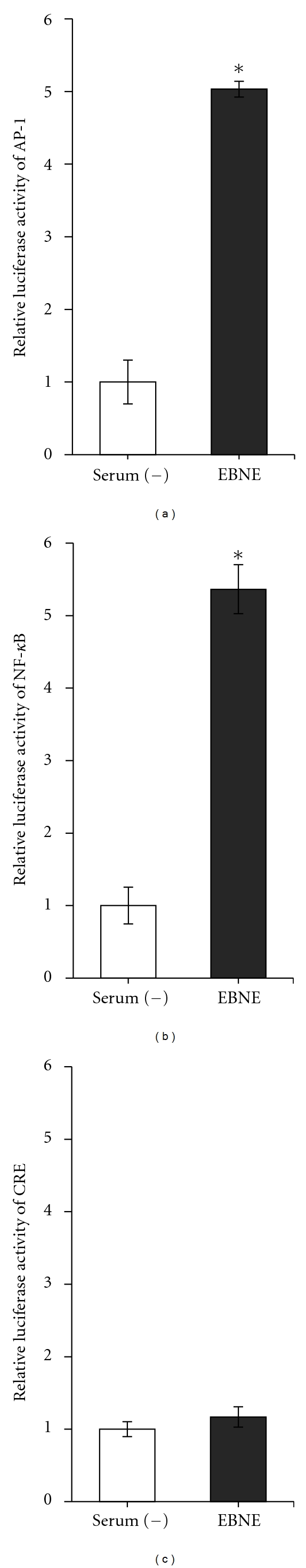
Effects of EBNE on AP-1, NF-*κ*B, or CRE promoter. hADSCs were transfected with AP-1 (a), NF-*κ*B (b), or CRE (c) luciferase reporters. After transfection, the cells were cultured with 2,000 ppm EBNE for 24 h, and then the luciferase assays were performed. Values are expressed as relative fold increase in luciferase activity compared with the untreated controls. Results are shown as mean ± SD (*n* = 3). *P < 0.05 versus untreated controls. Data are representative of at least three independent experiments. Serum (−): serum-free, EBNE: 2,000 ppm of EBNE.

**Figure 4:**
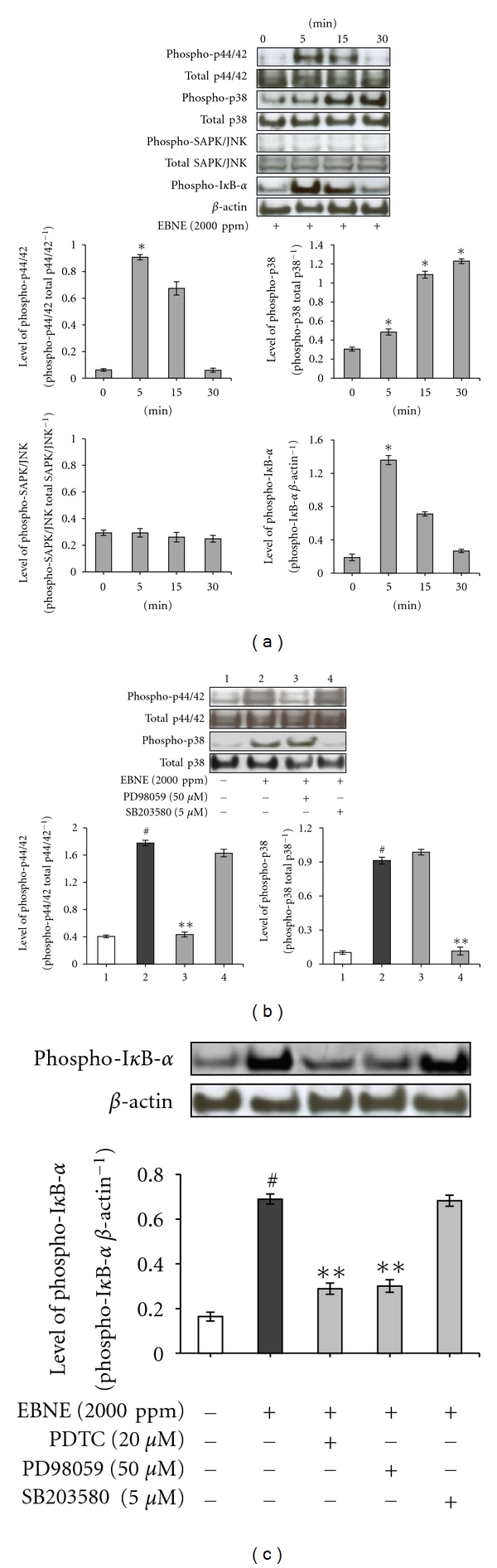
Effects of EBNE on MAPKs. (a) hADSCs were starved for 24 h and treated with 2,000 ppm EBNE for the indicated times. Total cell lysate was then prepared at 0, 5, 15, and 30 min following EBNE stimulation and subjected to Western blot analysis. The bands for phospho-p44/42, phospho-p38 MAPK, phospho-SAPK/JNK, and phosphor-I*κ*B-*α* were detected and normalized to their total form and *β*-actin. Results are shown as mean ± SD. *P < 0.05 versus untreated controls (0 time point). Data are representative of at least three independent experiments. (b, c) hADSCs were starved for 24 h and then treated with 50 *μ*M PD98059 or 5 *μ*M SB203580 or 20 *μ*M PDTC in the presence of EBNE. The cell lysate was prepared at 5 min (upon treatment of PD98059, PDTC) and 30 min (upon treatment of SB203580) and then subjected to Western blot analysis. The bands for phospho-p44/42, phospho-p38 MAPK, and phosphor-I*κ*B-*α* were detected and normalized to their total form and *β*-actin. Results are shown as mean ± SD. ^#^ *P* < 0.05 versus untreated controls. **P < 0.05 versus EBNE (2,000 ppm) only. Data are representative of at least three independent experiments.

AP-1 and NF-*κ*B regulate a variety of cellular genes that are important in the maintenance of cellular physiology [19, 20]. To elucidate which signaling pathways were involved in EBNE-induced expression of IL-6 and VEGF genes, luciferase reporter assays for AP-1, NF-*κ*B and CRE were performed in hADSCs. In this study, we found that EBNE induced activation of AP-1 and NF-*κ*B promoters, but not the CRE promoter (Figure 3), suggesting that AP-1 and NF-*κ*B signaling affect EBNE-induced expression of IL-6 and VEGF. To further investigate the mechanisms behind these EBNE effects, Western blot analysis for the phosphorylated forms of p44/42 MAPK, p38 MAPK, JNK, and I*κ*B-*α* was performed. As shown in Figure 4(a), while phosphorylation of p44/42 MAPK, p38 MAPK, and I*κ*B-*α* was induced by EBNE, no significant changes in JNK phosphorylation were observed. In addition, EBNE-induced phosphorylation of p44/42 MAPK, p38 MAPK, and I*κ*B-*α* was reduced by PD98059 (a p44/42 inhibitor), SB203580 (a p38 inhibitor), and PDTC (a NF-*κ*B inhibitor), respectively (Figures 4(b) and 4(c)). Furthermore, I*κ*B-*α* phosphorylation was reduced by PD98059, but were not affected by SB203580, suggesting that p44/42 MAPK also contributes to NF-*κ*B activation (Figure 4(c)).

### 3.4. EBNE-Induced Production of IL-6 and VEGF and Proliferation of hADSCs Are Regulated by AP-1 Signaling and NF-*κ*B Signaling

**Figure 5:**
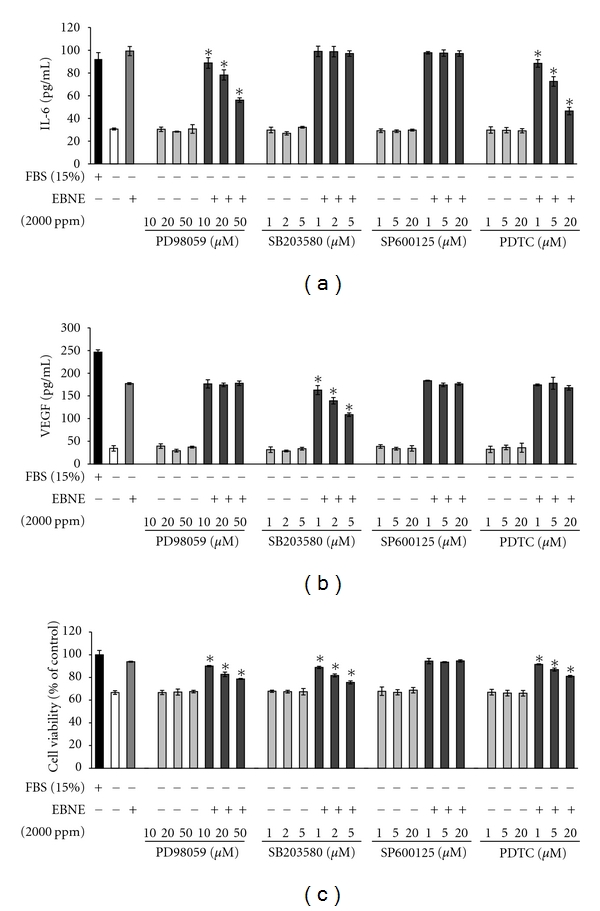
Effects of various inhibitors on the proliferation and cytokine expression in hADSCs. hADSCs were pretreated with the indicated concentrations of MAPK inhibitors (PD98059, SB203580, SP600125) or NF-*κ*B inhibitor (PDTC) for 3 h and then further incubated in the presence of 2,000 ppm EBNE. IL-6 and VEGF production was analyzed by ELISA (a, b). Cell proliferation was measured using the MTT assay (c). Results are shown as mean ± SD (*n* = 3). *P < 0.05 versus EBNE-treated controls. Data are representative of at least three independent experiments.

To examine the involvement of AP-1 signaling and NF-*κ*B signaling in EBNE-induced production of IL-6 and VEGF and proliferation of hADSCs, ELISA for IL-6 and VEGF and the MTT assay were performed. As shown in Figures 5(a) and 5(b), while EBNE-induced production of IL-6 was reduced by PD98059 (a p44/42 inhibitor) and PDTC (a NF-*κ*B inhibitor), it was not reduced by SB203580 (a p38 inhibitor) and SB203580 only suppressed EBNE-induced production of VEGF. These results indicate that EBNE-induced expression of IL-6 is mediated through the activation of p44/42 MAPK and NF-*κ*B, and VEGF expression is only induced by p38 MAPK activation. In addition, EBNE-induced proliferation of hADSCs was suppressed by PD98059 (a p44/42 inhibitor), SB203580 (a p38 inhibitor), and PDTC (a NF-*κ*B inhibitor), respectively (Figure 5(c)). Taken together, these results indicate that EBNE promotes proliferation of hADSCs by inducing expression of IL-6 and VEGF through the activation of NF-*κ*B and AP-1.

### 3.5. Mesenchymal Stem Cell Properties of EBNE-Induced hADSCs Are Maintained

**Figure 6:**
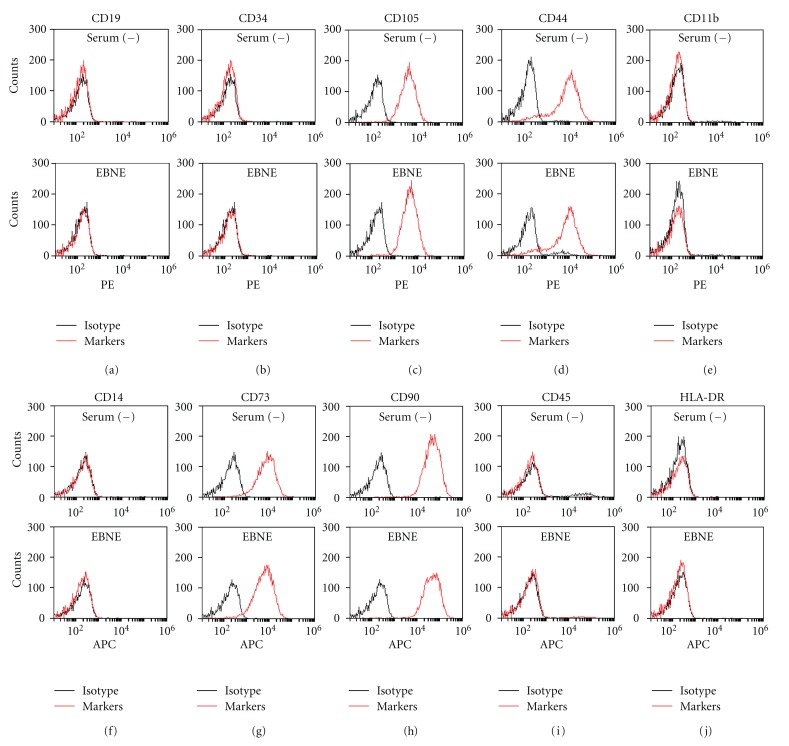
FACS analysis of surface markers of hADSCs. Cells were cultured in serum-free DMEM in the presence or absence of EBNE. The cells were then phenotyped using flow cytometry. (c), (d), (g), and (h) represent the positive mesenchymal stem cell (MSC) markers and (a), (b), (e), (f), (i), and (j) represent negative MSC markers. Red histograms show MSC specific markers, whereas isotype controls are represented in the black histograms. Serum (−): serum free, EBNE: 2,000 ppm of EBNE. Data are representative of at least two independent experiments.

Although EBNE was found to promote the proliferation of hADSCs, effects of EBNE on the mesenchymal stem cell properties of hADSCs are unknown. To examine this, FACS analysis was performed using several mesenchymal stem cell (MSC) markers. As shown in Figure 6, no significant changes in MSC markers between the serum-free group and EBNE-treated group were observed. These results indicate that EBNE promotes proliferation of hADSCs without impacting the mesenchymal stem cell properties of hADSCs.

## 4. Discussion

In this study, we report novel findings in regards to the proliferation of hADSCs induced by EBNE. We showed that (1) EBNE induces expression of IL-6 and VEGF; (2) induced expression of IL-6 and VEGF promotes proliferation of hADSCs; (3) EBNE-induced expression of IL-6 is regulated by activation of p44/42 MAPK and NF-*κ*B; (4) VEGF expression is induced by EBNE through the activation of p38 MAPK. Therefore, we demonstrated that EBNE promotes the proliferation of hADSCs by upregulating expression of IL-6 and VEGF through the activation of NF-*κ*B and AP-1 (p44/42 MAPK and p38 MAPK).

Stimulation with cytokines appears to play a crucial role in important normal cellular processes [21]. IL-6 has been shown to activate the JAK/STAT, MAPK, and AKT pathways and has been implicated as potent mediators of numerous important biological processes, including differentiation, apoptosis and proliferation [7]. In addition, VEGF has been shown to promote cell proliferation in response to hypoxia in human adipose stromal cells [12]. It has also been shown that VEGF production is mediated by a p38 MAPK-dependent mechanism [22]. In this study, we found that IL-6 and VEGF played an important role in the EBNE-induced proliferation of hADSCs. Therefore, our current work, combined with recent reports, supports a cell proliferation mechanism that is dependent on the expression of IL-6 and VEGF.

The MAPK and NF-*κ*B pathways, which involve a series of protein kinase cascades, play a critical role in the regulation of several changes in cell function such as cytokine expression, proliferation, and apoptosis [23–26]. Therefore, we hypothesized that the MAPK and NF-*κ*B pathways are involved in the EBNE-induced expression of IL-6 and VEGF. Inhibitors for MAPKs and NF-*κ*B were used to investigate the involvement of MAPKs and NF-*κ*B in the EBNE-induced proliferation of hADSCs. In mammalian cells, three MAPK cascades have been clearly characterized: p44/42 MAPK, p38 MAPK, and C-Jun SAPK/JNK [27]. In addition, three inhibitors for MAPKs were developed. PD98059 inhibits MEKs, an upstream activator of p44/42, which blocks p44/42-mediated signaling [28], SB203580 inhibits the p38 MAPK [29], and SP600125 inhibits SAPK/JNK [30]. In our study, while expression of IL-6 gene was found to be dependent on the activation of p44/42 MAPK and NF-*κ*B, p38 MAPK only played a central role in the expression of VEGF. Consistent with this, EBNE-induced proliferation of hADSCs was reduced by inhibitors for p44/42 MAPK, p38 MAPK, and NF-*κ*B, demonstrating that MAPKs and NF-*κ*B promote proliferation of hADSCs through the upregulation of IL-6 and VEGF.

 A stem cell is defined functionally as a cell with the capacity for self-renewal, as well as the ability to generate multiple differentiated cell types. Due to their capacity for proliferation and specialization, combined with their active self-renewal activity, stem cells are highly unique [14, 31–34]. Thus, given their expected capacity for self-renewal, hADSCs have been of great interest. The present study explored the ability of EBNE to improve the self-renewal function of hADSCs via an increase of the proliferative capacity. We found that EBNE enhanced stem cell potency via an increase in the proliferative capacity of hADSCs, suggesting that EBNE can act as an extracellular factor that improves self-renewal via an increase in the proliferative capacity of hADSCs. In addition, although the EBNE was shown to affect normal human cells, EBNE showed no significant effects on the transformed cell lines (MCF-7 and Hep2B). These results indicate that EBNE contributed to the increased proliferation of hADSCs and NHFs, but not human cancer cell lines, suggesting that the effects of EBNE are limited to normal cells and do not affect transformed cell lines.

In this study, we evaluated the proliferation-inducing effect and the possible mechanisms by which EBNE improves the proliferation of hADSCs. Collectively, the results of this study indicate that AP-1 (p44/42 MAPK and p38 MAPK) and NF-*κ*B-dependent production of IL-6 and VEGF plays a crucial role in the EBNE-induced proliferation of hADSCs under serum-free conditions.

##  Conflict of Interests

The authors declare that there is no conflict of interests.

## Figures and Tables

**Table 1:** Amino acid distribution (mg/g) of edible bird's nest extract.

Name	Total amino acid (T)	Flee amino acid (F)	(F/T) × 100
Aspartic acid	40.44	0.08	0.19
Threonine	22.39	1.32	5.89
Serine	29.47	0.84	2.85
Glutamic acid	51.78	0.27	0.52
Proline	21.07	0	0
Glycine	18.34	1.77	9.65
Alanine	18.44	2.79	15.13
Cysteine	41.06	0.06	0.14
Valine	24.35	8.88	36.46
Methionine	5.77	5.07	87.86
Isoleucine	16.65	8.36	50.21
Leucine	26.06	14.19	54.45
Tyrosine	17.16	11.83	68.93
Phenylalanine	29.37	16.19	55.12
Histidine	16.54	5.26	31.80
Lysine	15.23	6.08	39.92
Arginine	18.36	4.78	26.03
Tryptophan	0	6.02	100
